# Prevalence of physical health conditions and health risk behaviours in people with severe mental illness in South Asia: multi-country cross-sectional survey

**DOI:** 10.1192/bjo.2023.12

**Published:** 2023-02-23

**Authors:** Gerardo A. Zavala, Asiful Haidar-Chowdhury, Krishna Prasad-Muliyala, Kavindu Appuhamy, Faiza Aslam, Rumana Huque, Humaira Khalid, Pratima Murthy, Asad T. Nizami, Sukanya Rajan, David Shiers, Najma Siddiqi, Kamran Siddiqi, Jan R. Boehnke

**Affiliations:** Department of Health Sciences, University of York, UK; ARK Foundation, Dhaka, Bangladesh; Department of Psychiatry, National Institute of Mental Health and Neurosciences, Bangalore, India; Institute of Psychiatry, Rawalpindi Medical University, Pakistan; Psychosis Research Unit, Greater Manchester Mental Health NHS Trust, Manchester, UK; Division of Psychology and Mental Health, University of Manchester, UK; and School of Medicine, Keele University, UK; Department of Health Sciences, University of York, UK; Hull York Medical School, UK; and Bradford District Care NHS Foundation Trust, Bradford, UK; Department of Health Sciences, University of York, UK; and Hull York Medical School, UK; Department of Health Sciences, University of York, UK; and School of Health Sciences, University of Dundee, UK

**Keywords:** Physical health conditions, health risk behaviour, multimorbidity, comorbidity, severe mental illness

## Abstract

**Background:**

People with severe mental illness (SMI) die earlier than the general population, primarily because of physical disorders.

**Aims:**

We estimated the prevalence of physical health conditions, health risk behaviours, access to healthcare and health risk modification advice in people with SMI in Bangladesh, India and Pakistan, and compared results with the general population.

**Method:**

We conducted a cross-sectional survey in adults with SMI attending mental hospitals in Bangladesh, India and Pakistan. Data were collected on non-communicable diseases, their risk factors, health risk behaviours, treatments, health risk modification advice, common mental disorders, health-related quality of life and infectious diseases. We performed a descriptive analysis and compared our findings with the general population in the World Health Organization (WHO) ‘STEPwise Approach to Surveillance of NCDs’ reports.

**Results:**

We recruited 3989 participants with SMI, of which 11% had diabetes, 23.3% had hypertension or high blood pressure and 46.3% had overweight or obesity. We found that 70.8% of participants with diabetes, high blood pressure and hypercholesterolemia were previously undiagnosed; of those diagnosed, only around half were receiving treatment. A total of 47% of men and 14% of women used tobacco; 45.6% and 89.1% of participants did not meet WHO recommendations for physical activity and fruit and vegetable intake, respectively. Compared with the general population, people with SMI were more likely to have diabetes, hypercholesterolemia and overweight or obesity, and less likely to receive tobacco cessation and weight management advice.

**Conclusions:**

We found significant gaps in detection, prevention and treatment of non-communicable diseases and their risk factors in people with SMI.

## Severe mental Illness

Severe mental illnesses (SMIs) are conditions such as schizophrenia, bipolar disorder and psychotic depression that are debilitating, persistent and associated with serious functional impairment. People with SMI die on average 10–20 years earlier than the general population, and this ‘mortality gap’ is widening.^1^ Although suicide accounts for 15% of deaths, an estimated 80% of the observed premature mortality is attributable to physical disorders (physical multimorbidity), most commonly non-communicable diseases (NCDs).^2^

## Physical health in people with SMI

The excess disease burden from physical multimorbidity in people with SMI may be explained by a combination of factors associated with these mental disorders, including clustering of and predisposition to health risk behaviours (e.g. tobacco and alcohol use, lack of physical activity and poor diet), side-effects of medication, social determinants of poor health (e.g. stigma and poverty) and barriers to accessing healthcare.^3^ Our current understanding of the distribution and determinants of physical multimorbidity in people with SMI is based mostly on evidence from high-income countries. A few small studies from low- and middle-income countries (LMICs) show similar patterns, but with an even shorter life expectancy and higher mortality for people with SMI.^4,5^ These studies indicate that physical multimorbidity in SMI may be at least as much of a challenge in LMICs as in high-income countries.^1^

In South Asia, the prevalence of both mental disorders and NCDs has been increasing rapidly.^6^ This increase is coupled with limited access to essential health services and a widespread neglect of the physical health needs of people with SMI by policy makers and healthcare services.^7^ The overall burden of disease resulting from physical multimorbidity in this population is, therefore, likely to be high and is set to rise further, with a corresponding increase in within-country and global health inequalities. Despite these concerns, there is a lack of empirical studies originating in South Asia on the distribution and determinants of physical multimorbidity in people with SMI.^8^

Addressing multimorbidity in LMICs is a global priority, recognised in global policies to help achieve the United Nations Sustainable Development Goals.^9^ A detailed understanding of the prevalence of physical multimorbidity and current access to health advice and treatments for physical disorders in people with SMI in LMICs can inform appropriate service provision and contribute to achieving these goals.

## Aims

In the current study, we aim to (a) estimate the prevalence of physical health conditions and health risk behaviours; (b) assess access to physical healthcare and health risk modification advice in people with SMI attending mental health services in Bangladesh, India and Pakistan; and (c) compare the findings with those of the general population.

## Method

We conducted a cross-sectional survey of patients with a clinical diagnosis of SMI recruited at three national specialist mental health institutions in South Asia: the National Institute of Mental Health in Dhaka, Bangladesh; the National Institute of Mental Health and Neurosciences in Bangalore, India and the Institute of Psychiatry in Rawalpindi, Pakistan. Further details of the methods are reported in the published protocol,^10^ and are summarised below.

### Sample size

We aimed to build as large a sample as possible within the resources available over the study period, with an initial target of 1500 participants at each site. As an indicative example of precision to address some of the key research questions, we used the example of diabetes. For investigating the prevalence of type 2 diabetes, assuming a prevalence estimate of 10%, 857 participants per country would provide a precision of ±2% (95% confidence interval).

### Eligibility

Consenting adults (aged ≥18 years) with a clinical diagnosis of SMI as defined by the ICD-10 (schizophrenia, schizotypal and delusional disorders (F20–F29); bipolar affective disorder (F30, F31) or severe depression with psychotic symptoms (F32.3, F33.3)), and able to provide informed consent as assessed by the treating clinician, were eligible.

### Confirmation of SMI diagnosis

To increase standardisation across sites and alignment with other studies, each SMI diagnosis was confirmed by trained researchers using the Mini-International Neuropsychiatric Interview (MINI) version 6.0.^11^ The MINI is a short diagnostic structured interview for mental disorders, designed to allow administration by non-specialists.

### Recruitment of participants

We used stratified random sampling to recruit a sample comprising 80% out-patients and 20% in-patients. This reflects the flow of in- and out-patients in the three mental health hospitals on any given day, which was assessed and protocolised in each site before the data collection.

### Patient and public involvement

A community panel comprising patients, caregivers and advocacy group members ensured community, patient and public involvement. The panel reviewed and piloted the planned survey questionnaire, and advised on its feasibility.

### Data collection

We conducted a face-to-face survey with tablets (Qualtrics, Utah, USA; https://www.qualtrics.com/) to collect information about physical disorders, mental health, health risk behaviours, health-related quality of life, health risk behaviour advice and healthcare utilisation, using, wherever available, validated instruments as described below. The survey was translated into Bangla, Hindi, Kannada and Urdu. Interviewers (including males and females, to accommodate participant preference) used regional dialects where required, consistent with usual clinical practice in these settings. Data were collected between July 2019 and December 2021.

### STEPwise Approach to Surveillance of NCDs

We used the World Health Organization (WHO) STEPwise Approach to Surveillance of NCDs (STEPS) instrument version 3.2 to collect information about NCDs, associated risk factors and behaviours, access to physical healthcare and health risk modification advice.^12^ STEPS is an international standardised tool that has already been translated, used and validated in the general population in Bangladesh, India and Pakistan, and therefore allows comparisons with the general population within and between countries.^13,14^ The STEPS survey includes the use of show cards with culturally relevant examples used to aid respondents in classifying health risk behaviours. Categorisation of health conditions and risk behaviours followed the WHO guidelines.^15^

The STEPS module for NCDs was used to ask participants about medically diagnosed type 2 diabetes, raised blood pressure, heart disease and hypercholesterolemia, and treatments advised by a healthcare worker for these conditions (such as medication and dietary, weight management, smoking cessation or physical activity advice). Questions about lung disease, hepatitis B and C, syphilis, tuberculosis and HIV (which are not part of the STEPS survey) were asked in the same format as for the other chronic physical conditions.

### Health risk behaviours

Current or past use of smoking or smokeless tobacco was recorded.^15^ The alcohol module was used to categorise participants into lifetime abstainers, abstainers in the past 12 months and current users of alcohol;^15^ and the diet module was used to record the number of days that respondents consumed fruit and vegetables in a typical week, the number of servings consumed on average per day, and adherence to the WHO recommendations of at least five fruits and vegetables per day.^16^ The physical activity module was used to record activity for transport purposes (e.g. walking, cycling), vigorous and moderate activity at work, vigorous and moderate activity in leisure time and time spent sitting. In addition, risk behaviours related to sexually transmitted diseases, including multiple sexual partners, unprotected sexual contact and use of injectable drugs, were assessed with three questions adapted from the ten-item HIV Risk Screening Instrument.^17^

### Physical measurements

Blood pressure was taken with an automated blood pressure measuring instrument (OMRON) following instructions in the WHO STEPS surveillance manual; the average of the second and third readings was used for analysis.^15^ High blood pressure was defined as a measurement of >140/90 mmHg.^15^

Height, weight and waist circumference were measured for all participants except pregnant women. All measurements were taken in duplicate and the average of the two values was calculated, following the protocols set out in the WHO STEPS surveillance manual.^15^ We calculated the body mass index (BMI) and classified participants according to the WHO classification: underweight (BMI < 18.49 kg/m^2^), normal weight (BMI = 18.5–24.9 kg/m^2^), overweight (BMI = 25–29.9 kg/m^2^) or obese (BMI ≥ 30 kg/m^2^). Abdominal obesity was defined as a waist circumference of ≥94 cm for males and ≥80 cm for females.^15^

### Mental health

In addition to administering the MINI, we collected information relevant to the SMI diagnosis, including duration of illness and type and duration of treatments. The Patient Health Questionnaire-9 (PHQ-9) was used to measure the severity of depressive symptoms, and the Generalised Anxiety Disorder-7 (GAD-7) for severity of anxiety symptoms.

### Health-related quality of life

The EQ-5D-5L was used to measure health-related quality of life.^18^ We used the English, Urdu and Bangla validated versions, provided by EuroQol.

### Blood tests

A blood sample was taken from consenting participants for haemoglobin, glycated haemoglobin (HbA1c), lipid profile, thyroid function tests, liver function tests and creatinine. The cut-off for high HbA1c was according to the WHO definition of ≥6.5%.^19^ The prevalence of high total triglycerides was defined as ≥180 mg/dL,^20^ high serum cholesterol was defined as a low-density lipoprotein cholesterol of ≥100 mg/dL.^20^ Anaemia was defined as haemoglobin ≤13 g/dL for males and ≤12 g/dL for females.^21^

### Statistical analysis

Quantitative data were summarised with descriptive statistics, with mean, s.d. and 95% confidence interval for continuous data and counts, and percentage and 95% confidence interval for categorical data. Overall and by site, we described the prevalence of chronic physical conditions; prevalence of risk factors (obesity, high blood pressure and hypercholesterolemia) and risk behaviours (poor diet, physical inactivity, tobacco and alcohol use); severity of common mental disorder symptoms (anxiety, depression) and health-related quality of life; and access to treatment for physical conditions and health risk modification advice.

To compare our findings with those in the latest STEPS reports from Bangladesh,^22^ India^23^ and Pakistan,^14^ we calculated weights by comparing the gender and age distribution reported in these STEPS surveys with the distribution in our data. Because of the multiple differences within countries in the operationalisation of socioeconomic status and the definition of rural and urban populations, we did not weight our sample for sociodemographic and geographic variables.

Weights were applied with the complex sample module in SPSS version 26.0 for Windows, and we calculated the odds of people with SMI having an NCD, related risk factors, engaging in health risk behaviours, being screened, being treated and receiving risk modification advice compared with the STEPS survey participants in Bangladesh, India and Pakistan,^14,22,23^ using Stata version 17.0 for Windows. Results were presented as odds ratios from cross-tabulations of STEPS and weighted survey data. Significance levels were adjusted via Bonferroni correction for multiple hypothesis testing (adjusted level *P* < 0.006).

### Ethics statement

Trained researchers provided verbal and written study information to patients and their relatives or caregivers, highlighting that participation was voluntary, the decision would not affect care and consent could be withdrawn at any stage without providing a reason. Written consent was obtained (a thumbprint was accepted where a signature could not be provided). No assessments were conducted where the patient appeared reluctant, even if consent had previously been obtained. The study was approved by the ethics committees of the Department of Health Sciences, University of York, UK (HSRGC-3/17); the Centre for Injury Prevention and Research, Bangladesh (CIPRB/ERC/2OI 8/003); the Institute Ethics Committee, National Institute of Mental Health and Neurosciences, India (BEH.SC.DIV 20/19); the Health Ministry Screening Committee, India (HMSC12/18); and the National Bioethics Committee, Pakistan (4-18/NBC-413/19). All study procedures complied with legislation and guidance for good practice governing the participation in research of people who may lack capacity (Mental Capacity Act (UK) 2005). Participants did not receive financial inducements to participate, but results of physical health measurements and blood tests were shared with them and with the treating clinician. This study is registered with the ISRCTN registry under identifier ISRCTN88485933 (https://doi.org/10.1186/ISRCTN88485933). All participants consented and signed an informed consent form.

## Results

**Fig. 1:**
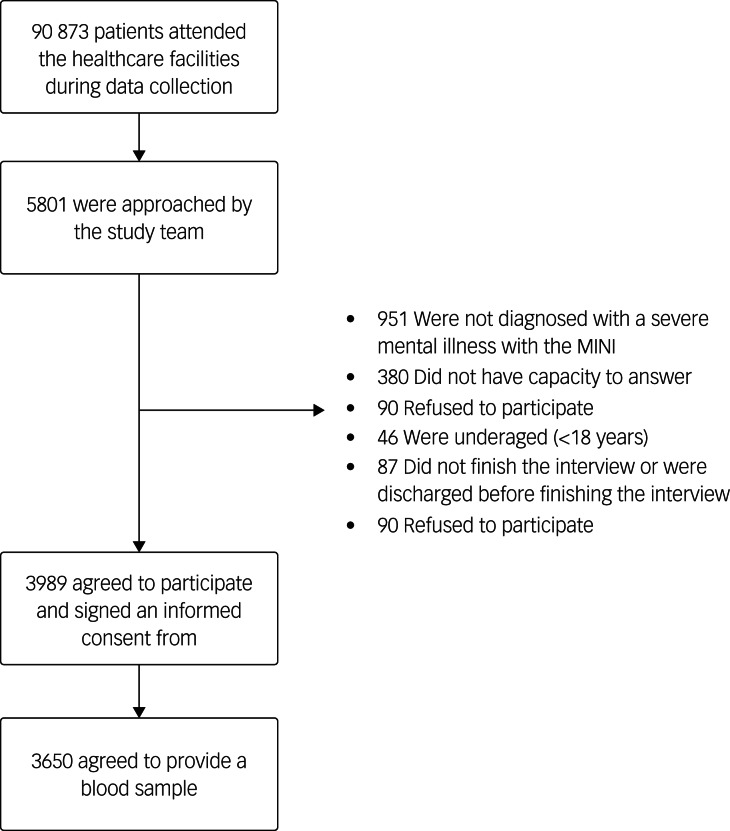
Participant flow chart. MINI, Mini-International Neuropsychiatric Interview.

We approached 5801 people with SMI in the three sites and 3989 (58.8%) participated in the survey (1500 in Bangladesh, 1175 in India and 1314 in Pakistan). Most of the participants in Bangladesh (94%) and Pakistan (70%) were recruited before the COVID-19 pandemic (July 2019 and March 2020), and most of the participants in India (86%) were recruited after the COVID-19 had begun (February 2021–Dec 2021). The details of participants that were not eligible are provided in Figure 1.

Participant characteristics are shown in Table 1. The proportion of in-patients was 20% for Bangladesh and India, and 10% for Pakistan. In-patients were more likely to be ineligible because of not having the capacity to sign an informed consent or answer the questionnaire. On average, 60.1% of the sample was male, and the mean age was 35.8 years; the Bangladesh cohort was younger than the cohort in India and Pakistan. Almost a third (32.0%) were unemployed, with a higher proportion in Bangladesh (39.7%) than India (19.4%) and Pakistan (22.2%). About half (50.8%) were educated only up to or less than primary school level.

**Table 1:** General characteristics of the participants

	Bangladesh (*n* = 1500)	India (*n* = 1175)	Pakistan (*n* = 1314)	Overall (*N* = 3989)
	*n* (%) [95% CI]	*n* (%) [95% CI]	*n* (%) [95% CI]	*n* (%) [95% CI]
General characteristics				
Gender (female)	585 (39.0) [36.6–41.5]	527 (44.9) [42.0–47.7]	518 (39.4) [36.8–42.1]	1630 (40.9) [39.3–42.4]
Age (years),^a^ mean (s.d.)	31.5 (10.8) [31.0–32.1]	38.8 (11.2) [38.1–39.4]	38.1 (12.3) [37.4–38.8]	35.8 (11.4) [35.5–36.2]
Education				
No formal education	151 (10.1) [8.6–11.7]	141 (12.0) [10.3–14.0]	257 (19.6) [17.5–21.8]	549 (13.8) [12.7–14.9]
Primary	842 (56.1) [53.6–58.6]	401 (34.1) [31.5–36.9]	234 (17.8) [15.8–20]	1477 (37.0) [35.6–38.5]
Secondary/higher	507 (33.8) [31.4–36.2]	632 (53.8) [50.9–56.6]	821 (62.5) [59.8–65.1]	1960 (49.1) [47.6–50.6]
Refused to answer^b^	(<1%)	(<1%)	(<1%)	(<1%)
Monthly household income over past year, USD,^a^ mean [95% CI]	224 [206–242]	305 [252–357]	198 [187–209]	237 [221–253]
Cement/concrete roof	427 (28.5) [26.2–30.8]	751 (63.9) [61.1–66.6]	1034 (78.8) [76.5–80.9]	2212 (55.5) [54.1–56.9]
Electricity in the household	1465 (97.7) [96.8–98.3]	1171 (99.7) [99.1–99.9]	1292 (100.0) [Not applicable]	3928 (99.0) [99.7–99.3]
Flush toilet in the household	866 (57.7) [55.2–60.2]	1046 (89) [87.1–90.7]	1269 (100.0) [Not applicable]	3181 (80.7) [79.5–81.7]
Occupation^b^				
Non-government employee	153 (10.2) [8.8–11.8]	207 (17.6) [15.5–19.9]	377 (28.7) [26.3–31.2]	737 (18.5) [17.3–19.7]
Government employee	18 (1.2) [0.8–1.9]	44 (3.7) [2.8–5.0]	53 (4.0) [3.1–5.2]	115 (2.9) [2.4–3.4]
Self-employed	252 (16.8) [15.0–18.8]	271 (23.1) [20.7–25.6]	68 (5.2) [4.1–6.5]	591 (14.8) [13.8–15.9]
Retired^b^	(<2%)	(<2%)	(<2%)	35 (0.9) [0.6–1.2]
Non-paid^b^	(<2%)	(<2%)	(<2%)	25 (0.6) [0.4–0.9]
Student	118 (7.9) [6.6–9.3]	42 (3.6) [2.7–4.8]	25 (1.9) [1.3–2.8]	185 (4.6) [4–5.3]
Homemaker	345 (23.0) [20.9–25.2]	371 (31.6) [29.0–34.3]	466 (35.5) [32.9–38.1]	1182 (29.6) [28.2–31.1]
Unemployed (able to work)	301 (20.1) [18.1–22.2]	137 (11.7) [9.9–13.6]	268 (20.4) [18.3–22.7]	706 (17.7) [16.6–18.9]
Unemployed (unable to work)	294 (19.6) [17.7–21.7]	90 (7.7) [6.3–9.3]	23 (1.8) [1.2–2.6]	407 (10.2) [9.3–11.1]
Did not wish to answer^b^	(<1%)	(<1%)	(<1%)	(<1%)
Currently married/living with partner	818 (54.5) [52.0–57.0]	711 (60.5) [57.7–63.3]	747 (56.8) [54.2–59.5]	2276 (57.1) [55.5–58.6]
Severe mental illness (MINI)				
Bipolar disorder	488 (32.5) [30.2–34.9]	464 (39.5) [36.7–42.3]	537 (40.9) [38.2–43.6]	1489 (37.3) [35.8–38.8]
Non-psychosis to affective psychosis	935 (62.3) [59.8–64.8]	673 (57.3) [54.4–60.1]	176 (13.4) [11.6–15.3]	1784 (44.7) [43.3–46.1]
Major depressive disorder with psychotic features	77 (5.1) [4.1–6.4]	66 (5.6) [4.4–7.1]	605 (46.0) [43.4–48.7]	748 (18.8) [17.7–19.8]
Patient setting				
In-patient	313 (20.9) [18.9–23.0]	264 (22.5) [20.2–24.9]	122 (9.3) [7.8–11]	699 (17.5) [16.4–18.7]
Out-patient	1187 (79.1) [77–81.1]	911 (77.5) [75.1–79.8]	1192 (90.7) [89.0–92.2]	3290 (82.5) [81.3–83.6]
Duration of SMI				
≤2 years	436 (29.1) [26.8–31.4]	215 (18.3) [16.2–20.6]	289 (22.0) [19.8–24.3]	940 (23.6) [22.3–24.9]
3–5 years	457 (30.5) [28.2–32.8]	266 (22.6) [20.3–25.1]	320 (24.4) [22.1–26.7]	1043 (26.1) [24.8–27.5]
6–10 years	332 (22.1) [20.1–24.3]	299 (25.4) [23.0–28]	299 (22.8) [20.6–25.1]	930 (23.3) [22.0–24.7]
>10 years	271 (18.1) [16.2–20.1]	359 (30.6) [28.0–33.3]	399 (30.4) [27.9–32.9]	1029 (25.8) [24.5–27.2]
Do not know or do not remember	(<1%)	(<4%)	(<1%)	(<2%)
On antipsychotic medication	1463 (97.5) [96.6–98.2]	1150 (97.9) [96.9–98.6]	1253 (95.4) [94.1–96.4]	3866 (96.9) [96.3–97.4]
Mental health				
Severity of depressive symptoms				
PHQ-9 score^a^	10.7 (4.6) [10.4–10.9]	5.8 (6.8) [5.4–6.2]	12.9 (6.8) [12.5–13.2]	10.0 (6.1) [9.8–10.2]
None or minimal (0–4)	121 (8.1) [6.8–9.6]	665 (56.6) [53.7–59.4]	177 (13.5) [11.7–15.4]	963 (24.1) [23–25.3]
Mild (5–9)	487 (32.5) [30.1–34.9]	224 (19.1) [16.9–21.4]	271 (20.6) [18.5–22.9]	982 (24.6) [23.3–26]
Moderate or severe (≥10)	892 (59.5) [57.0–61.9]	286 (24.3) [22.0–26.9]	866 (65.9) [63.3–68.4]	2044 (51.2) [49.8–52.7]
Severity of anxiety symptoms				
GAD-7 score^a^	8.1 (3.9) [7.9–8.3]	4.6 (5.4) [4.2–4.9]	9.9 (5.1) [9.6–10.2]	7.6 (4.8) [7.5–7.8]
None or minimal (0–4)	275 (18.3) [16.5–20.4]	729 (62.0) [59.2–64.8]	201 (15.3) [13.4–17.3]	1205 (30.2) [28.9–31.5]
Mild (5–9)	703 (46.9) [44.3–49.4]	236 (20.1) [17.9–22.5]	431 (32.8) [30.3–35.4]	1370 (34.3) [32.9–35.8]
Moderate or severe (≥10)	522 (34.8) [32.4–37.2]	210 (17.9) [15.8–20.2]	682 (51.9) [49.2–54.6]	1414 (35.4) [34.2–36.9]
Health-related quality of life				
Visual analogue scale^a^	69.9 (13.4) [69.2–70.6]	76.4 (17.6) [75.4–77.4]	54.9 (26.3) [53.4–56.3]	66.9 (19.7) [66.3–67.5]
Mobility	471 (31.4) [29.1–33.8]	252 (21.4) [19.2–23.9]	708 (53.9) [51.2–56.6]	1431 (35.9) [34.5–37.3]
Self-care	526 (35.1) [32.7–37.5]	230 (19.6) [17.4–21.9]	618 (47.0) [44.3–49.7]	1374 (34.4) [33.0–35.9]
Usual activities	684 (45.6) [43.1–48.1]	364 (31.0) [28.4–33.7]	727 (55.3) [52.6–58]	1775 (44.5) [43.0–46]
Pain/discomfort	993 (66.2) [63.8–68.6]	443 (37.7) [35.0–40.5]	1006 (76.6) [74.2–78.8]	2442 (61.2) [59.8–62.6]
Anxiety/depression	1363 (90.9) [89.3–92.2]	511 (43.5) [40.7–46.3]	959 (73.0) [70.5–75.3]	2833 (71.0) [69.7–72.3]

Confidence intervals were calculated using bootstrap sampling procedure (*n* = 1000) for binomial and continuous variables and Goodman's method for multinomial proportions. MINI, Mini-International Neuropsychiatric Interview; SMI severe mental illness; PHQ-9, Patient Health Questionnaire-9; GAD-7, Generalised Anxiety Disorder-7.

a. Values presented as mean (s.d.).

b. Data not reported because of low numbers for statistical disclosure control.

### SMI, anxiety and depressive symptoms and health-related quality of life

The most common SMI diagnosis was non-affective psychosis (44.7%), followed by bipolar disorder (37.3%) and depression with psychotic symptoms (18.8%). Non-affective psychosis was the most common diagnosis in Bangladesh (62.3%) and India (57.3%), whereas depression with psychotic symptoms was the most common diagnosis in Pakistan (46.0%). Almost 97% of participants were on antipsychotic medication.

A majority of participants reported having depressive (75.8%) and anxiety (69.7%) symptoms in the ‘moderate or severe’ category. The prevalence of ‘moderate or severe’ depressive symptoms was lower in India (24.3%) than in Bangladesh (59.5%) and Pakistan (65.9%). Similarly, a smaller proportion reported ‘moderate or severe’ anxiety symptoms in India (17.9%) than Bangladesh (34.8%) and Pakistan (51.9%).

The mean EQ-5D-5L (health-related quality of life) visual analogue scale (0–100) score was 66.9 overall, 69.9 for Bangladesh, 76.4 for India and 54.9 for Pakistan. A total of 45% of the participants reported problems in carrying out their usual activities; and around 60% reported pain/discomfort (Table 1).

### Physical disorders, risk factors, health risk behaviours and healthcare

As seen in Table 2, 11% of participants had type 2 diabetes (self-report of clinician diagnosis or those with an HBA1c >6.5%), 1% had chronic respiratory disorders, 3.2% had cardiovascular diseases, 2.3% had tuberculosis and 2.0% had chronic hepatitis.

**Table 2:** Non-communicable and communicable diseases and health risk behaviours in people with severe mental illness

	Bangladesh (*n* = 1500)	India (*n* = 1175)	Pakistan (*n* = 1314)	Overall (*N* = 3989)
	*n* (%) [95% CI]	*n* (%) [95% CI]	*n* (%) [95% CI]	*n* (%) [95% CI]
Non-communicable diseases				
Type 2 diabetes diagnosed by HbA1c test (≥6.50%) or self-report of clinician diagnosis^a^	127 (8.8) [7.4–10.3]	164 (16.1) [14.0–18.5]	121 (9.4) [7.9–11.1]	412 (11.0) [10.0–12.0]
Type 2 diabetes (self-report of clinician diagnosis only)	42 (2.8) [2.1–3.8]	94 (8.0) [6.6–9.7]	76 (5.8) [4.6–7.2]	212 (5.3) [4.7–6.1]
Heart disease (angina or a stroke) (self-report of clinician diagnosis)	20 (1.3) [0.9–2.1]	33 (2.8) [2–3.9]	73 (5.6) [4.4–6.9]	126 (3.2) [2.7–3.7]
Lung condition (self-report of clinician diagnosis)^b^	(<3%)	(<3%)	(<3%)	41 (1.0) [0.8–1.4]
Non-communicable disease risk factors				
High blood pressure (≥140/90 mmHg) or self-report of clinician diagnosis^c^	242 (16.1) [14.4–18.1]	241 (20.6) [18.3–23.0]	444 (33.8) [31.3–36.4]	927 (23.3) [22.0–24.6]
High blood pressure (self-report of clinician diagnosis only)	89 (5.9) [4.8–7.2]	85 (7.2) [5.9–8.9]	293 (22.3) [20.1–24.6]	467 (11.7) [10.8–12.7]
Hypercholesterolemia (tested plus self-report of clinician diagnosis)^d^	659 (46.0) [43.4–48.5]	470 (47.5) [44.4–50.6]	716 (56.0) [53.3–58.7]	1845 (49.9) [48.2–51.5]
Hypercholesterolemia (self-report of clinician diagnosis only)	14 (0.9) [0.6–1.6]	37 (3.1) [2.3–4.3]	53 (4.0) [3.1–5.2]	104 (2.6) [2.2–3.1]
Body mass index, kg/m²^e^	24.0 (4.5) [23.8–24.2]	25.6 (5.3) [25.3–25.9]	26.1 (7.0) [25.7–26.4]	25.2 (5.7) [24.9–25.3]
Body mass index categories				
Underweight (<18.5)	129 (8.6) [7.3–10.2]	66 (5.7) [4.5–7.2]	113 (8.7) [7.2–10.3]	308 (7.8) [7.0–8.6]
Normal weight (18.5–24.9)	817 (54.6) [52.0–57.1]	505 (43.5) [40.7–46.4]	498 (38.2) [35.6–40.8]	1820 (45.9) [44.4–47.5]
Overweight (25–29.9)	405 (27.1) [24.9–29.4]	384 (33.1) [30.4–35.8]	404 (31.0) [28.5–33.5]	1193 (30.1) [28.7–31.5]
Obese (≥30)	146 (9.8) [8.3–11.4]	206 (17.7) [15.6–20.1]	290 (22.2) [20.0–24.6]	642 (16.2) [15.1–17.4]
Waist circumference, cm^e^	83.7 (11.6) [83.1–84.3]	89.8 (22.9) [88.5–91.2]	90.7 (27.8) [89.2–92.2]	87.8 (21.4) [87.1–88.5]
High waist circumference, women (≥80 cm)	393 (67.5) [63.6–71.2]	380 (73.6) [69.7–77.3]	374 (73.8) [69.8–77.4]	1147 (71.5) [69.2–73.6]
High waist circumference, men (≥94 cm)	128 (14.0) [11.9–16.4]	247 (38.6) [34.9–42.4]	298 (37.9) [34.6–41.4]	673 (28.7) [27.0–30.6]
Communicable diseases				
Hepatitis B or hepatitis C (self-report of clinician diagnosis)^b^	(<5%)	(<5%)	(<5%)	78 (2.0) [1.6–2.4]
Tuberculosis (self-report of clinician diagnosis)^b^	(<5%)	(<5%)	(<5%)	92 (2.3) [1.9–2.8]
HIV (self-report of clinician diagnosis only)^b^	(<1%)	(<1%)	(<1%)	(<1%)
Physical activity				
Time spent on vigorous physical activity, min/day^e^	69.7 [15.4–22.5]	48.7 [10.3–15.8]	76.4 [15.3–23.6]	66.8 [15.3–19.4]
Vigorous physical activity (>0 min/day)	196 (13.1) [11.5–14.9]	181 (15.4) [13.4–17.6]	178 (13.5) [11.8–15.5]	555 (13.9) [12.9–15.0]
Time spent on moderate physical activity, min/day^e^	32.1 [28.2–34.8]	31.2 [25.3–37.3]	19.5 [17.0–25.1]	28.8 [25.5–30.6]
Moderate physical activity (>0 min/day)	600 (40.0) [37.5–42.5]	289 (24.6) [22.2–27.1]	289 (22.0) [19.8–24.3]	1178 (29.5) [28.2–30.9]
Time spent walking/cycling, min/day^e^	34.5 [30.3–38.7]	9.5 [7.7–11.3]	27.4 [23.4–31.3]	24.8 [22.7–26.9]
Cycling/walking activity (>0 min/day)	896 (59.7) [57.2–62.2]	273 (23.2) [20.9–25.7]	792 (60.3) [57.6–62.9]	1961 (49.2) [47.7–50.6]
Prevalence of low physical activity (total physical activity MET mins per week <600)	889 (59.3) [56.8–61.7]	389 (33.1) [30.5–35.9]	541 (41.2) [38.5–43.9]	1819 (45.6) [44.1–47.1]
Diet				
Do not meet WHO recommendations (fewer than five servings of fruits or vegetables per day)	1262 (84.1) [82.2–85.9]	1122 (95.5) [94.1–96.5]	1171 (89.1) [87.3–90.7]	3555 (89.1) [88.1–90]
Tobacco use				
Currently smoke, men (daily)	392 (42.8) [39.7–46.1]	130 (20.1) [17.2–23.3]	252 (31.7) [28.5–35]	774 (32.8) [31–34.7]
Currently smoke, women (daily)^b^	(<5%)	(<5%)	(<5%)	33 (2.0) [1.4–2.8]
Currently use smokeless tobacco, men	148 (16.2) [13.9–18.7]	118 (18.2) [15.4–21.4]	325 (40.8) [37.5–44.3]	591 (25.1) [23.4–26.8]
Currently use smokeless tobacco, women	112 (19.1) [16.2–22.5]	52 (9.9) [7.6–12.7]	49 (9.5) [7.2–12.3]	213 (13.1) [11.5–14.8]
Any form of tobacco, men	460 (50.3) [47–53.5]	201 (31) [27.6–34.7]	442 (55.5) [52.1–59]	1103 (46.8) [44.8–48.7]
Any form of tobacco, women	112 (19.1) [16.2–22.5]	55 (10.4) [8.1–13.4]	68 (13.1) [10.5–16.3]	235 (14.4) [12.8–16.2]
Risk behaviours for infectious diseases				
Two or more sexual partners in the past 10 years	137 (9.1) [7.8–10.7]	19 (1.6) [1.0–2.5]	197 (15.2) [13.3–17.2]	353 (8.9) [8.1–9.8]
Used condom when having sex in the past 10 years (among those who responded yes for two or more sexual partners)				
Never (report denominators) (*n* = 353)	26 (19.0) [13.2–26.5]	4 (21.1) [7.9–45.4]	119 (60.4) [53.4–67]	149 (42.2) [37.6–47]
Sometimes or always (*n* = 353)	111 (81.0) [73.5–86.8]	15 (78.9) [54.6–92.1]	78 (39.6) [33–46.6]	204 (57.8) [53–62.4]
Ever injected street drugs, steroids or vitamins with a needle^b^	(<1%)	(<1%)	39 (3.0) [2.2–4.1]	52 (1.3) [1.0–1.7]

Confidence intervals were calculated using bootstrap sampling procedure (*n* = 1000) for binomial and continuous variables and Goodman's method for multinomial proportions. HbA1C, glycated haemoglobin; MET, metabolic equivalents; WHO, World Health Organization.

a. The denominators for HbA1c are 2267 overall, 1370 for Bangladesh and 897 for Pakistan.

b. Data not reported because of low numbers for statistical disclosure control.

c. The denominators for hypertension are 2343 overall, 1422 for Bangladesh and 921 for Pakistan.

d. The denominators for hypercholesterolemia are 2247 overall, 1357 for Bangladesh and 890 for Pakistan.

e. Values presented as mean (s.d.).

Overall, 46.3% of participants were overweight or obese; most women (71.5%) and a high proportion of men (28.7%) had a high waist circumference. Underweight was also prevalent in 7.8% of the participants.

Almost a quarter (23.3%) either reported a diagnosis of hypertension or had high measured blood pressure (>140/90 mmHg): 16.1% in Bangladesh, 20.6% in India and 33.8% in Pakistan. Almost half (49.9%) were found to have hypercholesterolemia based on either previous reported diagnosis or high levels of low-density lipoprotein cholesterol. A total of 35% of participants had anaemia; this was higher in Bangladesh (44.9%, 95% CI 42.3–47.4%) than India (31.6%, 95% CI 28.7–34.5%) and Pakistan (28.3%, 95% CI 25.9–30.8%) (Supplementary Material available at https://doi.org/10.1192/bjo.2023.12). Most people with hypercholesterolemia (94.4%) and almost half with diabetes (49.2%) and with high measured blood pressure (48.5%) were previously unaware of their condition and were detected during the survey through cholesterol, HbA1c and blood pressure measurements, respectively.

Almost half of men (46.8%) consumed either smoking or smokeless tobacco, and 32.8% reported smoking tobacco daily. Smoking rates were 42.8% in Bangladesh, 20.1% in India and 31.7% in Pakistan. A total of 19% of women reported using tobacco in Bangladesh, 10.4% in India and 13.1% in Pakistan. Around half of participants (45.6%) did not meet the WHO recommendations for physical activity (of 600 metabolic equivalents); and 89.1% of the participants reported to not meet the WHO recommended levels of fruit and vegetable intake (at least five servings). Less than 6.2% of males and 0.4% of females reported consuming alcohol in the past month (data not provided in the tables). Less than 9% of the sample reported to have more than two sexual partners in the past 10 years.

As shown in Table 3, only 56.8% of the participants had been previously tested for any NCDs or NCD risk factors: 52.5% for hypertension, 26.7% for type 2 diabetes and 9.0% for hypercholesterolemia. In general, a low proportion of participants received treatment for physical conditions or to address risk factors. Of those with a self-reported NCD or an NCD risk factor, only 48.5% reported receiving related treatment or health risk modification advice. The provision of relevant treatment was highest in those reporting type 2 diabetes (74.5%, 95% CI 68.2–80.0%), followed by hypertension (43.9%, 95% CI 39.5–48.4%) and hypercholesterolaemia (34.6%, 95% CI 25.9–44.5%). Only 42.8% received any type of advice to modify health risk behaviours; the proportion of participants that received any type of health risk modification advice was highest in India (81.7%), followed by Pakistan (54.2%) and Bangladesh (23.8%). Among those who consumed tobacco, only 28.1% had been advised to quit.

**Table 3:** Proportion of people with severe mental illness screened, diagnosed and treated for non-communicable diseases and their risk factors, including health risk behaviour modification advice

	Bangladesh (*n* = 1500)	India (*n* = 1175)	Pakistan (*n* = 1314)	Overall (*N* = 3989)
	*n* (%) [95% CI]	*n* (%) [95% CI]	*n* (%) [95% CI]	*n* (%) [95% CI]
Screened, diagnosed and treated for NCDs and NCD risk factors (self-reported)				
Type 2 diabetes				
Ever had blood glucose measured by doctor or healthcare provider	334 (22.3) [20.2–24.4]	361 (30.7) [28.1–33.4]	372 (28.3) [25.9–30.8]	1067 (26.7) [25.4–28.1]
Ever diagnosed with type 2 diabetes by doctor or healthcare provider	42 (12.6) [9.4–16.6]	94 (26) [21.8–30.8]	76 (20.4) [16.6–24.8]	212 (19.9) [17.6–22.4]
Have received treatment for diabetes (among those with type 2 diabetes)^a^	30 (71.4) [55.8–83.2]	75 (79.8) [70.3–86.8]	53 (69.7) [58.4–79.1]	158 (74.5) [68.2–80.0]
Unaware of having type 2 diabetes (among those with type 2 diabetes)^a^	85 (66.9) [58.2–74.6]	70 (42.7) [35.3–50.4]	45 (37.2) [29–46.2]	200 (48.5) [43.9–53.2]
Hypertension				
Ever had blood pressure measured by doctor or healthcare provider	788 (52.5) [50–55.1]	506 (43.1) [40.3–45.9]	800 (60.9) [58.2–63.5]	2094 (52.5) [51–54]
Ever diagnosed with hypertension by doctor or healthcare provider	89 (11.3) [9.3–13.7]	85 (16.8) [13.8–20.3]	293 (36.6) [33.4–40]	467 (22.3) [20.6–24.1]
Have received treatment (among those diagnosed with hypertension)^a^	45 (50.6) [40.2–60.9]	47 (55.3) [44.6–65.6]	113 (38.6) [33.1–44.3]	205 (43.9) [39.5–48.4]
Unaware of having hypertension (among those diagnosed with hypertension)^a^	153 (63.2) [56.9–69.1]	156 (64.7) [58.5–70.5]	151 (34.0) [29.7–38.6]	460 (49.6) [46.5–52.7]
Hypercholesterolemia				
Ever had cholesterol measured by doctor or healthcare provider	61 (4.1) [3.2–5.2]	174 (14.8) [12.9–17]	126 (9.6) [8.1–11.3]	361 (9.0) [8.2–10]
Ever diagnosed with hypercholesterolemia by doctor or healthcare provider	14 (23.0) [14.0–35.3]	37 (21.3) [15.8–28]	53 (42.1) [33.7–50.9]	104 (28.8) [24.4–33.6]
Have received treatment (among those diagnosed with hypercholesterolemia)^a^^,^^b^	(<40%)	(<40%)	17 (32.1) [20.8–46]	36 (34.6) [25.9–44.5]
Unaware of having hypercholesterolemia (among those diagnosed with hypercholesterolemia)^a^	645 (97.9) [96.4–98.7]	433 (92.1) [89.3–94.2]	663 (92.6) [90.4–94.3]	1741 (94.4) [93.2–95.3]
Any NCD or NCD risk factor (type 2 diabetes, lung disease, heart disease, hypertension or hypercholesterolemia)				
Ever been tested for any NCD or NCD risk factor^c^	836 (55.7) [53.2–58.2]	574 (48.9) [46–51.7]	856 (65.1) [62.5–67.7]	2266 (56.8) [55.3–58.3]
Ever been diagnosed with any NCD or NCD risk factor^c^	132 (15.8) [13.5–18.4]	193 (33.6) [29.9–37.6]	380 (44.4) [41.1–47.7]	705 (31.1) [29.3–33]
Received treatment from doctor or other healthcare worker for any of these (among those with NCD or NCD risk factor)	69 (52.3) [43.7–60.7]	111 (57.5) [50.4–64.3]	162 (42.6) [37.7–47.7]	342 (48.5) [44.9–52.2]
Unaware of having any NCD or NCD risk factor (among those diagnosed) (denominators: Bangladesh 824, Pakistan 932, India 658, overall 2414)^a^	692 (84) [81.3–86.3]	465 (70.7) [67.1–74]	552 (59.2) [56–62.3]	1709 (70.8) [69–72.5]
Screened, diagnosed and treated for communicable diseases (self-reported)				
Ever been tested for hepatitis B or C	25 (1.7) [1.1–2.5]	96 (8.3) [6.8–10]	228 (17.4) [15.4–19.5]	350 (8.8) [8–9.7]
Diagnosed with hepatitis B or C among those tested^b^	(<25%)	(<8%)	65 (28.5) [23–34.7]	77 (22.0) [18–26.5]
Ever been tested for tuberculosis	36 (2.4) [1.7–3.3]	24 (2.0) [1.4–3]	190 (14.5) [12.7–16.5]	250 (6.3) [5.6–7]
Diagnosed with tuberculosis among those tested	(<52%)	(<26%)	68 (35.8) [29.2–42.9]	92 (36.8) [31–43]
Ever been tested for HIV	25 (1.7) [1.1–2.5]	71 (6.0) [4.8–7.6]	12 (0.9) [0.5–1.6]	108 (2.7) [2.3–3.3]
Received treatment for any chronic communicable disease (among those diagnosed with a chronic communicable disease)	56 (100.0) [100.0–100.0]	7 (58.3) [29.5–82.4]	137 (92.6) [87–95.9]	200 (92.6) [88.5–95.3]
Health risk behaviour advice				
Quit tobacco or do not start	127 (9.8) [8.3–11.5]	72 (17.1) [13.8–21]	102 (14.1) [11.7–16.8]	301 (12.3) [11.1–13.7]
Quit tobacco among those who currently smoke or use smokeless tobacco	108 (22.2) [18.7–26.1]	51 (49.5) [39.9–59.1]	86 (30.5) [25.4–36.1]	245 (28.1) [25.3–31.1]
Reduce salt in diet	74 (5.7) [4.6–7.1]	86 (20.4) [16.8–24.6]	138 (19.0) [16.3–22.1]	298 (12.2) [11–13.5]
Reduce salt in diet among those diagnosed with hypertension	32 (15.5) [11.1–21.1]	40 (39.2) [30.2–49.1]	110 (41.8) [36–47.9]	182 (31.8) [28.2–35.6]
Eat at least five servings of fruit and/or vegetables each day	213 (16.4) [14.5–18.5]	248 (58.9) [54.1–63.5]	238 (32.8) [29.5–36.3]	699 (28.6) [27–30.3]
Reduce fat in diet	116 (8.9) [7.5–10.6]	98 (23.3) [19.5–27.6]	192 (26.5) [23.4–29.8]	406 (16.6) [15.2–18.1]
Start or do more physical activity	142 (10.9) [9.4–12.8]	286 (67.9) [63.3–72.2]	177 (24.4) [21.4–27.7]	605 (24.8) [23.3–26.3]
Maintain a healthy body weight or lose weight	96 (7.4) [6.1–9]	187 (44.4) [39.7–49.2]	102 (14.1) [11.7–16.8]	385 (15.8) [14.5–17.2]
Maintain a healthy body weight or lose weight among those with overweight or obesity	50 (10.2) [7.8–13.2]	137 (59.1) [52.6–65.2]	83 (20.0) [16.5–24.2]	270 (23.8) [21.6–26.1]
Reduce sugary beverages in diet	74 (5.7) [4.6–7.1]	74 (17.6) [14.2–21.5]	90 (12.4) [10.2–15]	238 (9.7) [8.6–11]
Reduce sugary beverages in diet among those with type 2 diabetes	21 (17.9) [12–26]	38 (49.4) [38.3–60.5]	39 (47.6) [36.9–58.4]	98 (35.5) [30.3–41.1]
Any type of lifestyle advice	309 (23.8) [21.6–26.2]	344 (81.7) [77.7–85.1]	393 (54.2) [50.6–57.8]	1046 (42.8) [41.1–44.6]
Healthcare utilisation				
Visited a doctor or other healthcare worker in the past 12 months	1297 (86.5) [84.6–88.1]	421 (35.8) [33.1–38.6]	725 (55.2) [52.5–57.8]	2443 (61.2) [59.9–62.6]

Confidence intervals were calculated using bootstrap sampling procedure (*n* = 1000) for binomial and continuous variables and Goodman's method for multinomial proportions. NCD, non-communicable disease.

a. People that self-reported not to have type 2 diabetes, hypertension and hypercholesterolemia or had not been tested, but were positive on the test performed during the current survey.

b. Data not reported because of low numbers for statistical disclosure control.

c. Includes type 2 diabetes, hypertension and hypercholesterolemia.

### Comparison between people with SMI and the general population (STEPS survey)

The results for the comparisons between our data and country STEPS reports are summarised in Table 4.

**Table 4:** Odds of people with severe mental illness having non-communicable diseases, related risk factors and health risk behaviours and receiving healthcare screening and advice compared with the general population (severe mental illness data weighted^a^)

	Bangladesh				India				Pakistan			
	STEPS, yes/total	SMI, yes/total	Odds ratio [95% CI]	*P-*value^b^	STEP, yes/total	SMI, yes/total	Odds ratio [95% CI]	*P-*value^b^	STEPS, yes/total	SMI, yes/total	Odds ratio [95% CI]	*P-*value^b^
Non-communicable diseases												
Type 2 diabetes (diagnosed by HbA1c test (≥6.50%) or self-report of clinician diagnosis)^c^	586/7056	192/1441	1.7 [1.42–2.03]	<0.001	887/9540	163/1014	1.87 [1.55–2.25]	<0.001	N/A	117/1271	N/A	N/A
Type 2 diabetes (self-report of clinician diagnosis only)	417/8185	71/1491	0.93 [0.71–1.21]	0.589	410/9540	93/1173	1.92 [1.50–2.43]	<0.001	250/7358	71/1297	1.65 [1.24–2.17]	<0.001
Cardiovascular diseases (angina or a stroke) (self-report of clinician diagnosis)	819/8185	26/1491	0.16 [0.10–0.24]	<0.001	373/10 659	34/1173	0.82 [0.56–1.18]	0.284	363/7357	68/1297	1.07 [0.80–1.40]	0.637
Non-communicable disease risk factors												
Hypertension (measured in survey or self-report of clinician diagnosis)	1684/8019	313/1491	1.0 [0.87–1.15]	0.995	3038/10 586	242/1170	0.65 [0.56–0.75]	<0.001	N/A	420/1296	N/A	N/A
Hypertension (self-report of clinician diagnosis only)	1121/8185	149/1491	0.7 [0.58–0.84]	<0.001	836/10 586	86/1173	0.92 [0.72–1.16]	0.494	1096/7358	290/1297	1.65 [1.42–1.91]	<0.001
Hypercholesterolemia (measured in survey or self-report of clinician diagnosis)	2002/7049	704/1419	2.48 [2.21–2.79]	<0.001	N/A	467/988	N/A	N/A	N/A	683/1261	N/A	N/A
Hypercholesterolemia (self-report of clinician diagnosis only)	176/8185	20/1491	0.62 [0.37–1.0]	0.041	192/10 659	37/1173	1.78 [1.21–2.55]	0.001	110/7357	48/1297	2.53 [1.76–3.61]	<0.001
Underweight (BMI < 18.5 kg/m^2^)	1038/7985	122/1488	0.6 [0.49–0.73]	<0.001	1999/10 409	66/1159	0.25 [0.19–0.33]	<0.001	747/6613	130/1286	0.88 [0.72–1.08]	0.215
Overweight or obesity (BMI ≥ 25 kg/m^2^)	2068/7985	609/1488	1.98 [1.76–2.23]	<0.001	2717/10 409	592/1159	2.96 [2.61–3.35]	<0.001	2725/6613	674/1286	1.57 [1.39–1.77]	<0.001
High waist circumference, women (≥80 cm)	1687/4104	568/795	3.58 [3.03–4.25]	<0.001	N/A	384/521	N/A	N/A	N/A	507/725	N/A	N/A
High waist circumference, men (≥94 cm)	556/3784	125/693	1.28 [1.02–1.59]	0.024	N/A	247/632	N/A	N/A	N/A	209/548	N/A	N/A
Health risk behaviours												
Low physical activity (total physical activity MET mins per week <600)	999/8118	646/1491	5.45 [4.81–6.17]	<0.001	4402/10 659	783/1173	2.85 [2.51–3.25]	<0.001	2932/7064	812/1297	2.36 [2.08–2.67]	<0.001
Do not meet WHO recommendations (fewer than five servings of fruits or vegetables per day)	7318/8168	1257/1491	0.62 [0.53–0.73]	<0.001	10488/10 659	1120/1173	0.34 [0.25–0.48]	<0.001	6899/7339	1150/1297	0.5 [0.41–0.61]	<0.001
Smoking, men (daily)	1773/3804	299/693	0.87 [0.74–1.03]	0.092	1338/5818	128/640	0.84 [0.68–1.03]	0.086	876/3150	176/555	1.21 [0.99–1.47]	0.060
Smoking, women (daily)^d^	44/4381	Not reported	0.25 [0.03–0.95]	0.037	63/4841	4/533	0.57 [0.15–1.55]	0.277	177/4216	37/742	1.2 [0.81–1.73]	0.33
Smokeless tobacco use, men	1023/3804	142/693	0.70 [0.57–0.86]	<0.001	2124/5818	117/640	0.39 [0.31–0.48]	<0.001	312/3150	226/555	6.25 [5.06–7.71]	<0.001
Smokeless tobacco use, women	1231/4381	188/798	0.79 [0.66–0.94]	0.008	581/4841	51/533	0.78 [0.56–1.05]	0.098	198/4216	58/742	1.72 [1.25–2.35]	<0.001
Any form of tobacco use, men	2267/3804	367/693	0.76 [0.65–0.90]	0.001	2979/5818	199/640	0.43 [0.36–0.51]	<0.001	1121/3150	308/555	2.26 [1.87–2.72]	<0.001
Any form of tobacco use, women	1240/4381	188/798	0.78 [0.65–0.93]	0.006	629/4841	54/533	0.75 [0.55–1.02]	0.060	367/4216	85/742	1.36 [1.04–1.75]	0.016
Screening for non-communicable diseases and risk factors												
Type 2 diabetes												
Ever had blood glucose measured by doctor or healthcare provider	2054/8185	434/1491	1.23 [1.08–1.39]	0.001	2509/9540	360/1173	1.24 [1.08–1.42]	0.001	831/7358	360/1297	3.02 [2.61–3.49]	<0.001
Ever diagnosed with type 2 diabetes by doctor or healthcare provider	417/2054	71/434	0.77 [0.57–1.02]	0.060	410/2509	93/360	1.78 [1.36–2.32]	<0.001	250/831	71/360	0.57 [0.42–0.78]	<0.001
Have received treatment (among those with type 2 diabetes)	244/417	55/71	2.44 [1.32–4.71]	0.002	158/410	74/93	6.21 [3.54–11.29]	<0.001	186/250	51/71	0.88 [0.47–1.68]	0.664
Unaware of having type 2 diabetes (among those with type 2 diabetes)^e^	301/586	121/192	1.61 [1.14–2.29]	0.005	477/887	70/163	0.64 [0.45–0.91]	0.011	N/A	47/117	N/A	N/A
Hypertension												
Ever had blood pressure measured by doctor or healthcare provider	5738/8185	886/1491	0.62 [0.56–0.70]	<0.001	5505/10 586	507/1173	0.7 [0.62–0.79]	<0.001	4025/7358	811/1297	1.38 [1.22–1.56]	<0.001
Diagnosed with hypertension by doctor or healthcare provider (among those with blood pressure previously measured)	1121/5738	149/886	0.83 [0.69–1.01]	0.056	836/5505	86/507	1.14 [0.88–1.46]	0.288	1096/4025	290/811	1.49 [1.26–1.75]	<0.001
Have received treatment (among those with hypertension)	301/1121	88/149	3.9 [2.7–5.7]	<0.001	134/836	47/86	6.3 [3.9–10.3]	<0.001	580/1096	119/290	0.62 [0.47–0.81]	<0.001
Unaware of having hypertension (among those with hypertension)^e^	864/1684	164/313	1.04 [0.81–1.34]	0.723	2202/3038	157/242	0.7 [0.53–0.94]	0.011	N/A	130/420	N/A	N/A
Hypercholesterolemia												
Ever had cholesterol measured by doctor or healthcare provider	377/8185	75/1491	1.1 [0.84–1.42]	0.475	682/10 659	175/1173	2.57 [2.14–3.08]	<0.001	456/7357	118/1297	1.51 [1.22–1.88]	<0.001
Diagnosed with hypercholesterolemia by doctor or healthcare provider (among those with cholesterol previously measured)	176/377	20/75	0.42 [0.23–0.74]	0.001	192/682	37/175	0.68 [0.45–1.03]	0.062	110/456	48/118	2.16 [1.37–3.37]	<0.001
Have received treatment (among those diagnosed with hypercholesterolemia)^d^	71/176	Not reported	0.63 [0.19–1.87]	0.370	74/192	14/37	0.97 [0.43–2.11]	0.936	48/110	16/48	0.65 [0.30–1.38]	0.225
Unaware of having hypercholesterolemia (among those with hypercholesterolemia)^e^	1898/2002	684/703	1.97 [1.19–3.43]	0.006	N/A	430/467	N/A	N/A	N/A	636/683	N/A	N/A
Advice on health risk behaviours												
Quit or do not take up tobacco	807/3977	106/1281	0.35 [0.28–0.44]	<0.001	1865/10 659	71/420	0.96 [0.73–1.25]	0.754	1832/7356	74/707	0.35 [0.27–0.45]	<0.001
Quit tobacco among those who currently use smoke or smokeless tobacco	434/638	90/461	0.11 [0.08–0.15]	<0.001	522/2985	50/102	4.5 [3.0–6.9]	<0.001	419/823	61/216	0.38 [0.27–0.53]	<0.001
Start or do more physical activity	764/3977	149/1281	0.55 [0.46–0.67]	<0.001	N/A	286/420	N/A	N/A	2060/7356	176/707	0.85 [0.71–1.02]	0.078
Maintain a healthy body weight or lose weight	660/3977	111/1281	0.48 [0.38–0.59]	<0.001	N/A	187/420	N/A	N/A	1964/7356	105/707	0.48 [0.37–0.59]	<0.001

STEPS, STEPwise Approach to Surveillance of NCDs; SMI, severe mental illness; HbA1C, glycated haemoglobin;, N/A: Not available in the STEPS survey; BMI, body mass index; MET, metabolic equivalents; WHO, World Health Organization.

a. Data from the general population was extracted from the STEPS 2018 survey in Bangladesh and India, and 2014 survey in Pakistan; data from the SMI survey were weighted by age and gender according to the distribution of the STEPS report.

b. After Bonferroni correction for multiple testing, the *P* < 0.05 significance level was corrected to *P* < 0.006.

c. Blood glucose ≥126 mg/dL for the STEPS survey and HbA1c ≥6.50% for the SMI survey.

d. Data not reported because of low numbers for statistical disclosure control.

e. People that self-reported not to have the condition or had not been previously tested but tested positive in assessments performed for the current survey or the STEPS survey in Bangladesh.

#### Prevalence of NCDs and NCD risk factors

People with SMI in Bangladesh (odds ratio 1.7, 95% CI 1.4–2.0, *P* < 0.001) and India (odds ratio 1.8, 95% CI 1.5–2.2, *P* < 0.001) were more likely to have type 2 diabetes compared with the general population, and people with SMI in Bangladesh were more likely to have hypercholesterolemia (odds ratio 2.4, 95% CI 2.2–2.7, *P* < 0.001) compared with the general population. Blood samples were not collected in the STEPS survey in Pakistan and for cholesterol in India, therefore these comparisons are not available.

People with SMI were more likely to be overweight or obese (BMI > 25 kg/m^2^) compared with the general population (Bangladesh: odds ratio 1.9, 95% CI 1.7–2.2, *P* < 0.001; India: odds ratio 2.9, 95% CI 2.6–3.3, *P* < 0.001; Pakistan: odds ratio 1.5, 95% CI 1.3–1.7, *P* < 0.001). People with SMI in Bangladesh were less likely to be underweight (odds ratio 0.6, 95% CI 0.4–0.7, *P* < 0.001), but there were no differences in India and Pakistan.

#### Health risk behaviours

People with SMI were more likely not to meet recommendations for physical activity (Bangladesh: odds ratio 5.4, 95% CI 4.8–6.1, *P* < 0.001; India: odds ratio 2.8, 95% CI 2.5–3.2, *P* < 0.001; Pakistan: odds ratio 2.3, 95% CI 2.0–2.6, *P* < 0.001). However, they were less likely not to meet WHO recommendations for fruit and vegetable intake (Bangladesh: odds ratio 0.6, 95% CI 0.5–0.7, *P* < 0.001; India: odds ratio 0.3, 95% CI 0.2–0.4, *P* < 0.001; Pakistan: odds ratio 0.5, 95% CI 0.4–0.6, *P* < 0.001).

Men with SMI in Bangladesh (odds ratio 0.7, 95% CI 0.6–0.9, *P* = 0.001) and India (odds ratio 0.4, 95% CI 0.3–0.5, *P* < 0.001) were less likely to use tobacco products, whereas the opposite was found in Pakistan (odds ratio 2.2, 95% CI 1.8–2.7, *P* < 0.001).

#### Screening and diagnosis

Compared with the general population, people with SMI were more likely to be screened for type 2 diabetes in Bangladesh (odds ratio 1.2, 95% CI 1.0–1.3, *P* = 0.001), India (odds ratio 1.2, 95% CI 1.0–1.4, *P* = 0.001) and Pakistan (odds ratio 3.0, 95% CI 2.6–3.4, *P* < 0.001).

People with SMI in Bangladesh (odds ratio 0.6, 95% CI 0.5–0.7, *P* < 0.001) and India (odds ratio 0.70, 95% CI 0.6–0.7, *P* < 0.001) were less likely to be screened for hypertension, whereas the opposite was found in Pakistan (odds ratio 1.3, 95% CI 1.2–1.5, *P* < 0.001). Among those screened, people with SMI in Pakistan were more likely to have hypertension (odds ratio 1.4, 95% CI 1.2–1.7, *P* < 0.001), whereas no differences were found in Bangladesh and India.

Regarding hypercholesterolemia, people with SMI in India (odds ratio 2.5, 95% CI 2.1–3.0, *P* < 0.001) and Pakistan were more likely to be screened (odds ratio 1.5, 95% CI 1.2–1.8, *P* < 0.001), and those that were screened in Pakistan were more likely to have hypercholesterolemia (odds ratio 2.1, 95% CI 1.3–3.3, *P* < 0.001) than people in the general population. In Bangladesh, there was no difference in screening; however, those that were screened were less likely (odds ratio 0.4, 95% CI 0.2–0.7, *P* = 0.001) to have hypercholesterolemia than the general population.

#### Health risk modification advice

People with SMI were less likely to receive advice to quit or not take up tobacco in Bangladesh (odds ratio 0.3, 95% CI 0.2–0.4, *P* < 0.001) and Pakistan (odds ratio 0.3, 95% CI 0.2–0.4, *P* < 0.001), whereas no differences were found in India. A similar pattern was observed for receiving advice on maintaining healthy body weight (Bangladesh: odds ratio 0.4, 95% CI 0.3–0.5, *P* < 0.001; Pakistan: odds ratio 0.4, 95% CI 0.3–0.5, *P* < 0.001, Pakistan); this indicator was not available for the STEPS report in India.

## Discussion

This is the first multi-country study from South Asia to report on physical multimorbidity, health risk behaviours and access to related healthcare in people with SMI. We found a high prevalence of physical health conditions, primarily NCDs and related risk factors. We also found that people with SMI were more likely to have NCDs and NCD risk factors (overweight/obesity, hypertension, hypercholesterolemia) and engage in some health risk behaviours (tobacco use), but were less likely to receive risk modification advice than the general population. Many people with SMI in our sample reported that they had never been tested or screened for NCDs or NCD risk factors despite the well-established link between SMI and cardiometabolic conditions.^4,5^ Moreover a large proportion of people with type 2 diabetes, hypertension and hypercholesterolaemia had not been previously diagnosed, and these conditions were only detected on testing during the survey. Most had not received appropriate treatment and risk modification advice for their physical health. Therefore, even in the two major specialist mental health institutes included in our survey, most people with SMI failed to receive adequate screening, prevention and management of NCDs and NCD risk factors.

At the time of the survey, there were no policies or recommendations for people with SMI to attend or visit primary care. The difference within countries in terms of healthcare utilisation (visited a doctor or other healthcare worker in the past 12 months) is most likely a result of the differences in the timing of data collection: most of the sample in India was recruited during the COVID-19 pandemic, whereas most of the sample in Bangladesh and Pakistan was recruited before the COVID-19 pandemic. This finding is supported by a multicentre cross-sectional study in India reporting that people had 2.5 higher odds of not being able to access healthcare services during the COVID-19 pandemic compared with before the COVID-19 pandemic.^24^

The finding that people with SMI are more likely to have NCD risk factors compared with the general population extends previous findings in high-income countries and LMICs for risk factors such as obesity, hypercholesterolemia and decreased physical activity.^25,26^ Importantly, it should be noted that psychotropic medication might contribute to some of these adverse risks.^27^ Almost all survey participants were prescribed antipsychotics, which are associated with tiredness and sedation, an increased risk of obesity and adverse effects on glucose and lipid metabolism. The high prevalence of anaemia among participants is consistent with findings in people with SMI in other LMICs,^28^ and this has been associated with poor diet and side-effects of mood stabilisers.^28^

In Pakistan, we found a higher prevalence of tobacco use in people with SMI compared with the general population. This is consistent with other studies in people with SMI,^29^ where tobacco use has been associated with a greater susceptibility to addiction because of a higher subjective experience of reward and an attempt to self-medicate to mitigate anxiety and depressive symptoms.^30^ Unexpectedly, the opposite was found in Bangladesh and India. This may be because the STEPS survey for Bangladesh and India reported an unusually high estimate of the prevalence of tobacco use (in men, 70% for Bangladesh and 52% for India). The more reliable Global Adult Tobacco Survey^31^ for the same period reported a prevalence of 58% in Bangladesh and 43% in India in the same group, which is closer to the figures reported in our study.

The low observed prevalence of alcohol use in both men and women is similar to the STEPS survey reports,^14,22,23^ and is likely to be explained by religious proscription.

Despite the high prevalence of overweight/obesity, hypercholesterolemia, hypertension and tobacco use, health risk modification advice was provided to less than a quarter of people with SMI, and we found that the odds of receiving such advice was lower in people with SMI than in the general population in Bangladesh and Pakistan. Similar treatment gaps have been reported in high-income countries.^25^ Although psychiatrists are trained in motivational interviewing, there are attitudinal barriers that make mental health professionals reluctant to engage with patients about their tobacco use.^32^ Moreover, misconceptions about potential side-effects of tobacco cessation medication, unfounded fears of exacerbating depressive symptoms following quitting and low expectations of patients’ motivation or ability to stop smoking are additional barriers.^33^ On the other hand, there is high-quality evidence from high-income countries about both the effectiveness and cost benefits of smoking cessation interventions in people with SMI.^34^ Such approaches need to be adopted in South Asia, where tobacco use is common. Similarly, lifestyle interventions have shown promise to reduce weight and improve metabolic risk factors, and are recommended as an essential part of the management of SMI in these countries.^35^

An important study finding is the differences in the proportion of participants with moderate or severe depressive and anxiety symptoms within the countries (with the highest in Pakistan and lowest in India). This may be explained by the type of patient flow in each hospital: the National Institute of Mental Health and Neurosciences is a tertiary care, exclusive neuropsychiatric setting, and the proportion of patients with schizophrenia and bipolar disorders is likely to be higher compared with a general hospital psychiatry unit, whereas the proportion of patients with depression is higher in the Institute of Psychiatry in Rawalpindi, Pakistan, as compared with private mental health facilities. These differences may also be related to the higher proportion of participants in the depression with psychosis category in Pakistan.

The prevalence of tuberculosis was three times higher than in the general population. This is consistent with previous findings in LMICs and the clustering of tuberculosis risk factors reported in people with SMI.^36^ In contrast, the prevalence of HIV^37^ and hepatitis B and C^38^ were similar to those reported in the general population – a surprising finding considering the several risk factors for blood-borne viruses that have been reported to cluster in people with SMI.

Although most of the comparisons between people with SMI and the general population are in line with clinical expectations and previous findings,^26^ there were some anomalous results. These include the lower odds of people with SMI with a self-reported clinical diagnosis of type 2 diabetes, hypertension and hypercholesterolemia in Bangladesh. This may be because of ‘diagnostic overshadowing’, where the presence of a mental disorder means clinicians do not look for physical health problems, or failure to recall such diagnosis by patients. The lower education and socioeconomic levels for participants from Bangladesh (compared with India and Pakistan) may have contributed to the latter.^39^

We report findings from the first large-scale effort to document physical multimorbidity in people with SMI attending specialist services in three South Asian countries. We used standardised tools for data collection (i.e. STEPS, EQ-5D-5L, PHQ-9, GAD-7) that allowed us to compare our findings with those in the general population. Data were collected by trained researchers having experience of working with this population. Finally, we gathered objective data on physical conditions (including blood tests), and reported on both previously diagnosed and undiagnosed conditions.

Of the several limitations that need to be mentioned, the first is although we have used findings from studies in the general population to compare and discuss our findings, caution needs to be exercised in such comparisons, since our sample was collected from mental health hospitals and the analyses were only adjusted by gender and age and other. Moreover, we need to be mindful of the time lag between these studies, during which a number of parameters of interest might have changed.

Second, we relied on blood results from each mental health institution's laboratory, but we did not standardise these tests between laboratories. Third, there are methodological considerations that should be considered when making comparisons between the countries: recruitment in India was done during the COVID-19 pandemic, which needs to be considered when comparing the country estimates, since the pandemic might have affected the physical and mental health outcomes as well as healthcare utilisation of the SMI population in India; in-patients were more likely to be excluded because a ‘lack of capacity to answer’, and these patients with more severe SMI symptoms have also shown to have more physical health problems,^40^ which may be associated with an underestimation of physical ill conditions in our sample; there was a lower proportion of in-patients in Pakistan than in Bangladesh and India, and in-patients are known to have more severe mental health symptoms and are more likely to have physical health conditions,^40^ which might lead to an underestimation of the prevalence of mental and physical health conditions in Pakistan. Fourth, the information from some of the questions from the STEPS survey are easy to recall (e.g. diabetes diagnosis), whereas some other may be more difficult (e.g. time performing physical activity or receipt of health risk modification advice) and prone to recall bias. To the best of our knowledge, there is no information on the performance of the access to healthcare and health risk modification advice questions in the STEPS survey that could provide further information about the risk of bias. Fifth, since the sample was drawn from tertiary care, the findings may not be representative of the total SMI population in each country. However, unlike mental health services in high-income countries, tertiary care services in South Asian countries accept self-referral without the need for primary or secondary care referral, and often function as ‘the first port of call’ for people with SMI. They also attract patients from both urban and rural areas. Therefore, the study population is likely to be similar to the overall population of people with SMI in these countries.

The high prevalence of physical health conditions and health risk behaviours in SMI compared with the general population, and their underdetection even in specialist centres, merits attention to improve early identification, prevention and management, in line with international recommendations and guidance. Given many of these physical health monitoring and management guidelines for SMI are based on evidence from (and developed in) high-income countries, they may not necessarily be applicable to low-resource settings in LMICs. Our findings can help to identify and contextualise the priority areas for LMICs, and to develop more appropriate guidance for such settings. In view of challenging resource limitations, interventions to address health risk behaviours that are brief and delivered by non-specialist personnel need to be tested in these settings. Integration of physical healthcare with mental healthcare that has been envisioned at all levels of mental healthcare delivery needs to be actioned and scaled up.^8^ Representative community-based studies may further answer questions related to regional differences in physical health conditions and health risk behaviours.

In conclusion, people with SMI in South Asia have a high prevalence of NCDs, which may be attributable to the associated clustering of several health risk factors and behaviours in this population. There is an unmet need to address physical multimorbidity in people with SMI in South Asia. Policy makers and healthcare professionals working with people with SMI need to recognise the extent and importance of physical multimorbidity in this vulnerable group, and prioritise the prevention, screening and treatment of NCDs in people with SMI.

## Data Availability

The data-sets used and/or analysed during the current study are available from the corresponding author, G.A.Z., on reasonable request.

## Supplementary material

For supplementary material accompanying this paper visit http://doi.org/10.1192/bjo.2023.12. click here to view supplementary material
