# Supplementary material for: Prevalence of physical health conditions and health risk behaviours in people with severe mental illness in South Asia: multi-country cross-sectional survey
Source: BJPsych Open. 2023 Feb 23;9(2):e43. doi: 10.1192/bjo.2023.12 (PMC9970179; doi:10.1192/bjo.2023.12)
Supplement: Supplementary file 1 [file S2056472423000121sup001.docx]

This is an electronic appendix accompanying the paper “The physical health of people with severe mental illness in Bangladesh Pakistan and India: A cross-sectional survey”

**Section 1. Blood test investigation in people with SMI from South Asia**

| **Blood test investigation in people with SMI from South Asia** | | | | | | | |  |
| --- | --- | --- | --- | --- | --- | --- | --- | --- |
|  | Bangladesh | | India | | Pakistan | | Overall | |
|  | n | percentage [95% C.I.] | n | percentage [95% C.I.] | n | Mean (S.D.)[95% C.I.] | n | Mean (S.D.)[95% C.I.] |
| Glycated haemoglobin (HbA1c) (%) | 1429 | 5.5 (0.8) [5.4-5.5] | 947 | 5.8(1.4)[5.7-59] | 1274 | 5.3 (2.1) [5.2-5.4] | 3650 | 5.5 (1.5) [5.4-5.4] |
| Glycated haemoglobin (HbA1c ≥6.50%) * | 95 | 6.6 [5.5-8.1] | 118 | 12.5[10.5-14.7] | 70 | 5.5 [4.4-6.9] | 283 | 7.8 [6.9-8.7] |
| Haemoglobin g/dl for male | 877 | 14.2 (0.8) [14.1-14.2] | 537 | 14.6(1.6) [14.5-14.7] | 769 | 14.9 (3.5) [14.6-15.1] | 2183 | 14.5 (2.3) [14.4-14.6] |
| Haemoglobin g/dl for female | 552 | 10.7 (0.7) [10.6-10.7] | 455 | 12.4(1.5) [12.22-12.5] | 505 | 12.7 (3.7) [12.4-13.0] | 1512 | 11.8 (2.3) [11.8-12.0] |
| Haemoglobin ( ≤13 g/dl males ≤12 g/dl females ) * | 641 | 44.9 [42.3-47.4] | 313 | 31.6[28.7-34.5] | 360 | 28.3 [25.9-30.8] | 1314 | 35.6 [34.1-37.1] |
| **Lipid profile** |  |  |  |  |  |  |  |  |
| Triglycerides (mg/dl) | 1427 | 177.9 (101.0) [172.6-183.1] | 950 | 173.4(113.8) [166.2-180.7] | 1272 | 172.3 (91.9)[167.2-177.3] | 3649 | 174.7(101.5) [171.5-178.1] |
| High triglycerides (>180 mg/dl) * | 572 | 40.1 [37.6-42.7] | 331 | 34.8 [31.9-37.9] | 470 | 36.9 [34.3-39.6] | 1373 | 37.6 [36.1-39.2] |
| Total cholesterol (mg/dl) | 1429 | 177.6 (34.6) [175.7-179.4] | 980 | 175.5(77.2) [170.7-180.3] | 1273 | 194.5(65.1)[191.0-198.2] | 3682 | 183(59.3)  [181-184.8] |
| Total cholesterol (≥190 mg/dl) * | 474 | 33.2 [30.8-35.7] | 307 | 31.3 [28.5-34.3] | 637 | 50 [47.3-52.8] | 1418 | 38.5 [37-40.1] |
| HDL cholesterol (mg/dl) | 1429 | 43.9 (3.5) [43.7-44.1] | 973 | 39.5(9.9) [38.9-40.1] | 1270 | 47.8(14.2)[47.0-48.6] | 3672 | 44(10.05)[43.8-44.4] |
| HDL cholesterol (≤40 mg/dl for males and ≤50 for females)* | 589 | 41.2 [38.7-43.8] | 728 | 74.8[72-77.5] | 567 | 44.6[41.9-47.4] | 1884 | 51.3 [49.8-52.9] |
| LDL cholesterol (mg/dl) | 1429 | 98.2 (28.6) [96.7-99.6] | 951 | 99.0(33.4) [96.9-101.1] | 1270 | 107.1(39.3) [104.9-109.2] | 3650 | 101.5(33.9)[100.4-102.6] |
| LDL cholesterol ( ≥100 mg/dl)* | 654 | 45.8 [43.2-48.4] | 443 | 46.6 [43.4-49.8] | 694 | 54.6 [51.9-57.4] | 1791 | 49.1 [47.5-50.7] |
| **Thyroid function test** |  |  |  |  |  |  |  |  |
| TSH (uIU/ml) | 1429 | 2.3 (2.1) [2.2-2.4] | 987 | 4.0(8.1)  [3.5-4.5] | 1215 | 2.9 (17.1) [2-3.9] |  |  |
| TSH ( ≤0.35 uIU/mL)*‡ | 17 | 1.2 [0.7-1.9] | 15 | 1.5 [0.9-2.5] | 31 | 2.6 [1.8-3.6] | 63 | 1.7 [1.4-2.2] |
| TSH ( ≥4.50 uIU/ml)* | 94 | 6.6 [5.4-8.0] | 196 | 19.9 [17.5-22.5] | 77 | 6.3 [5.1-7.9] | 367 | 10.1 [9.2-11.1] |
| Calcium (mg/dl) | 1429 | 9.0 (0.9) [9.0-9.1] | - | - | 1268 | 9.9 (3.9) [9.7-10.1] | 2697 | 9.4 (2.7) [9.3-9.5] |
| Calcium (≤8.5 mg/dl) * | 536 | 37.5 [35.0-40.1] | - | - | 193 | 15.2 [13.3-17.3] | 729 | 27 [25.4-28.7] |
| Calcium ( ≥10.5 mg/dl)* | 38 | 2.7 [1.9-3.6] | - | - | 358 | 28.2 [25.8-30.8] | 396 | 14.7 [13.5-16] |
| **Liver function test** |  |  |  |  |  |  |  |  |
| Total bilirubin (mg/dl) | 1429 | 0.4 (0.1) [0.4-0.4] | 988 | 0.5(0.4)[0.5-0.5] | 1272 | 0.6 (1.8) [0.5-0.7] | 3689 | 0.5(1.1)[0.5-0.5] |
| Total bilirubin (≥1.2 mg/dl) *‡ | N/R | <5% | N/R | <5% | N/R | <5% | 94 | 2.5 [2.1-3.1] |
| AST (U/L) | 1429 | 33.8 (18.1) [32.8-34.7] | 988 | 23.0(14.4)[22.1 - 23.9] | 1271 | 37.3 (19.2) [36.2-38.3] | 3688 | 32.1 (17.6) [31.5-32.7] |
| AST (≥35U/l) * | 601 | 42.1 [39.5-44.6] | 87 | 8.8[7.2 - 10.7] | 578 | 45.5 [42.8-48.2] | 1266 | 34.3 [32.9-35.8] |
| ALT (U/L) | 1429 | 35.3 (20.5)[34.2-36.3] | 987 | 22.3(16.5)[21.2 - 23.3] | 1273 | 37.5 (27.1) [35.8-38.9] | 3689 | 32.6 (24.4) [31.8-33.3] |
| ALT ( ≤7 U/L) *‡ | - | - | N/R | <5% | N/R | <5% | 40 | 1.1[0.8-1.5] |
| ALT (≥56 U/l) * | 128 | (9.0) [7.6-10.6] | 42 | 4.3[3.2 - 5.7] | 188 | (14.8) [12.9-16.8] | 358 | 9.7 [8.8-10.7] |
| ALP (U/L) | 1429 | 108.6 (41.8) [106.4-110.8] | 991 | 90.9(29.4)[89.0 - 92.7] | 1271 | 112.5 (45.4) [110.-115.0] | 3691 | 105.-2 (40.2) [103.9-106.5] |
| ALP (≤41U/L) * | 22 | 1.5 [1-2.3] | 11 | 1.1[0.6 -2] | 14 | 1.1 [0.7-1.9] | 47 | 1.3 [1-1.7] |
| ALP (≥133U/L) * | 491 | 34.4 [31.9-36.9] | 75 | 7.6[6.1 - 9.4] | 290 | 22.8 [20.6-25.2] | 856 | 23.2 [21.9 -24.5] |
| **Total protein** |  |  |  |  |  |  |  |  |
| Albumin (g/dl) | 1429 | 4.4 (0.4) [4.3-4.4] | 980 | 4.6(4.0)[4.3-4.8] | 1272 | 4.7(13.3) [4.0-5.5] | 3681 | 4.6 (8.1) [4.3-4.8] |
| Albumin (≤4.5 g/dl) * | 1013 | 70.9 [68.5-73.2] | 521 | 53.2[50-56.3] | 697 | 54.8 [52-57.5] | 2231 | 60.6 [59-62.2] |
| Albumin (≥5.3 g/dl) * | 21 | 1.5 [1.2-2] | 15 | 1.5[0.9-2.5] | 79 | 6.2 [5-7.7] | 115 | 3.1 [2.6-3.7] |
| Globulin (g/dl) | 1429 | 3.6 (1.0) [3.6-3.7] | 976 | 3.0(0.5)[3.0-3.1] | 1268 | 4.1 (21.4) [3-5.3] | 3673 | 3.6 (12.6) [3.2-4.0] |
| Globulin (≤2.5 g/dl) * | 176 | 12.3 [10.7-14.1] | 119 | 12.2[10.3-14.4] | 239 | 18.8 [16.8-21.1] | 534 | 14.5 [13.4-15.7] |
| Globulin (≥3.5 g/dl) * | 702 | 49.1 [46.5-51.7] | 180 | 18.4[16.1-2.1] | 608 | 47.9 [45.2-50.7] | 1490 | 40.6 [39-42.1] |
| Albumin to globulin ratio | 1429 | 1.4 (0.4) [1.3-1.4] | 979 | 1.5(0.4)[1.5-1.56] | 1266 | 2.6 (15.0) [1.8-3.4] | 3674 | 1.8 (8.8) [1.6-2.1] |
| Albumin to globulin ratio (≤1.2 and ≥2.5 ) * | 643 | 45 [42.4-47.6] | 175 | 17.9 [15.6-20.4] | 633 | 50 [47.2-52.8] | 1451 | 39.5 [38-41] |
| **Renal function test** |  |  |  |  |  |  |  |  |
| Serum Urea (mg/dl) | 1429 | 26.0 (7.8) [25.6-26.4] | 113 | 18(8.4) [16.6-19.7] | 1272 | 28.9 (10.5) [28.4-29.3] | 2814 | 27.0 (9.1) [26.6-27.3] |
| Serum Urea (≥43 mg/dl) *‡ | N/R | <2% | N/R | <2% | 103 | 8.1 [6.7-9.7] | 132 | 4.7 [4-5.5] |
| Creatinine (mg/dl) | 1429 | 0.9 (0.2) [0.9-0.9] | 991 | 0.8(0.6) [0.8-0.9] | 1274 | 0.8 (0.6) [0.8-0.9] | 3694 | 0.9 (0.5) [0.8-0.9] |
| Creatinine (≥ 0.95 mg/dl females and ≥1.14 mg/dl males) * | 169 | 11.8 [10.3-13.6] | 59 | 6[4.6-7.6] | 158 | 12.4 [10.7-14.3] | 386 | 10.4 [9.5-11.5] |
| *Values presented as n (%), NA not available, tests were not performed.‡ Data not reported in some columns due to low numbers for statistical disclosure control. | | | | | | | |  |
